# XGBoost outperforms other machine learning models in diagnosing Sepsis-Associated Thrombocytopenia: a multicenter retrospective study

**DOI:** 10.3389/fmed.2026.1715551

**Published:** 2026-02-09

**Authors:** Busra Emir, Evrim Ozmen, Sukriye Miray Kilincer Bozgul, Caner Acar, Nur Soyer, Devrim Bozkurt, Kamil Gonderen, Mehmet Göktuğ Efgan

**Affiliations:** 1 Department of Biostatistics, Faculty of Medicine, Izmir Katip Celebi University, Izmir, Türkiye; 2 Department of Biostatistics, Institute of Health Sciences, Izmir Katip Celebi University, Izmir, Türkiye; 3 Department of Internal Medicine, Faculty of Medicine, Ege University, Izmir, Türkiye; 4 Department of Internal Medicine, Faculty of Medicine, Izmir Katip Celebi University, Izmir, Türkiye; 5 Department of Emergency Medicine, Faculty of Medicine, Izmir Katip Celebi University, Izmir, Türkiye

**Keywords:** artificial intelligence, classification, extreme Gradient Boosting, machine learning, performance metrics, Sepsis-Associated Thrombocytopenia

## Abstract

**Objective:**

Sepsis-associated thrombocytopenia is a frequent complication of sepsis and is associated with poor clinical outcomes. Accurate diagnosis remains challenging, and machine learning approaches may offer improved diagnostic performance. The objective of this study was to conduct a comparative assessment of the performance of machine learning models (Random Forest, Artificial Neural Networks, Extreme Gradient Boosting, and Naive Bayes) in diagnosing sepsis-associated thrombocytopenia and to evaluate their diagnostic performance based on predefined performance criteria.

**Methods:**

This retrospective cross-sectional study was conducted at two centers and utilized data from 1,447 sepsis patients extracted from electronic health records between January 2013 and December 2023. The dataset comprised demographic and clinical attributes together with laboratory test results. The data were partitioned into training and test sets in an 80:20 ratio. All models were trained on the training set, and 10-fold cross-validation was applied within the training set to assess internal performance consistency. Model performance was evaluated using accuracy, precision, F1 score, AUROC, and confusion matrix. The ten most significant variables were ranked using classifier-based feature importance and SHAP analysis.

**Results:**

Thrombocytopenia occurred in 772 (53.4%) of the 1,447 sepsis patients. Cross-validation results indicated that XGBoost exhibited superior performance, achieving an accuracy of 91.10% and an F1 score of 92.31%. ANN and RF achieved accuracies of 90.32% and 83.75%, with F1 scores of 91.51% and 85.32%, respectively. The AUROC values for cross-validation and test sets of RF, ANN, XGBoost, and Naive Bayes models exceeded 90%.

**Conclusion:**

Among the evaluated algorithms, the XGBoost model demonstrated superior performance, achieving an AUROC of 98.60% in cross-validation and 97.50% in the test set, indicating the strongest performance metrics within internal validation.

## Introduction

Sepsis is a significant life-threatening public health issue that leads to organ failure due to immune system disturbance caused by the dissemination of severe infection throughout the body (1, 2). Sepsis, defined by updated clinical criteria known as Sepsis 3, was revised in 2016 due to new discoveries post-2001 and serves as a benchmark in patient assessment (3). The World Health Organization’s 2020 global report indicated that sepsis was responsible for 1 in every 5 deaths globally, with roughly 49 million cases and 11 million sepsis-related fatalities reported in 2017 (4).

Thrombocytopenia is a condition characterized by a reduced platelet count, defined as less than 150 × 10^9/L, and may arise from various etiologies (1, 5). Thrombocytopenia resulting from sepsis is termed Sepsis-Associated Thrombocytopenia (1). Thrombocytopenia is frequently observed in ICU patients, with over 55% of those with sepsis exhibiting this condition, and over 30% of patients with sepsis-associated thrombocytopenia experiencing severe thrombocytopenia (defined as <50 × 10^9/L) (5). The platelet count is now included in the SOFA score (Sepsis-Related Organ Failure Assessment), which assesses the extent of organ dysfunction in critically ill patients (6). Severe inflammation in sepsis leads to abnormalities in platelet production and a significant drop in platelet count, with low platelet levels contributing to mortality (7).

Machine learning, which utilizes algorithms to learn from existing data sets and constructs prediction models by validating them, holds significant promise in medical and data analytics (8). Given the SAT patient and death rates, early diagnosis and intervention are crucial. Machine learning demonstrates potential, since multiple studies indicate superior performance compared to existing approaches in sepsis diagnosis and classification; nevertheless, research on machine learning in SAT diagnosis and risk classification is sparse (9).

This study aims to accurately assess disease prognosis by classifying diagnoses, identify the top ten significant features in disease diagnosis, and evaluate the performance of machine learning classification models to facilitate appropriate treatment, while recommending the optimal model to the literature through a retrospective cross-sectional study conducted at two centers.

## Methods

### Study design

The research was carried out as a two-center retrospective cross-sectional study utilizing data sourced from electronic health records at Izmir Katip Celebi University Ataturk Training and Research Hospital and Ege University Hospital from January 2013 to December 2023. The dataset comprised 1,736 patients, with 1,268 patients (73%) from Izmir Katip Celebi University Ataturk Training and Research Hospital and 468 patients (27%) from Ege University Hospital. The study collected demographic data, vital signs, and laboratory results from the electronic health records of 1,447 sepsis patients (772 with thrombocytopenia and 675 without) aged 18 years and older, who were admitted to the emergency department.

### Diagnostic criteria

The inclusion criteria were determined as patients aged 18 years and older who were admitted to the emergency department of Izmir Katip Celebi University Ataturk Training and Research Hospital and Sepsis patients older the age of 18 in the intensive care unit of Ege University were identified. The objective was to assess the overall features of emergency department patients diagnosed with sepsis, encompassing various age demographics and clinical histories, in a more thorough manner. In the multicenter research by Wang et al., which identified the risk variables influencing SAT, patients with hematological and other malignancies were included, and the malignancy variable was examined independently. In our research, these patients were included and not assessed on a variable basis (10).

Sepsis was defined according to Sepsis-3 criteria, requiring documented or suspected infection together with an increase of ≥2 points in the Sequential Organ Failure Assessment (SOFA) score. Sepsis-associated thrombocytopenia (SAT) was defined using explicit diagnostic criteria serving as the ground truth for machine learning classification. SAT was diagnosed when either of the following conditions was met a platelet count < 150 × 109/L, or a ≥ 50% decrease from the patient’s known baseline platelet count occurring during the sepsis episode. The diagnosis of SAT was confirmed by the attending clinicians based on complete blood count trends, organ dysfunction scores (including SOFA and APACHE II), inflammatory markers, hemodynamic parameters, and overall clinical evaluation. Patients with alternative etiologies of thrombocytopenia such as known chronic thrombocytopenia, hematologic malignancy with bone marrow failure, active chemotherapy, heparin-induced thrombocytopenia, or immune thrombocytopenia were excluded from SAT classification. These criteria reflect widely accepted definitions used in prior sepsis and thrombocytopenia research and ensure clinical interpretability of the machine learning outputs. These diagnostic criteria constituted the reference standard labels used for machine learning model training and testing ensuring consistency across both centers. All machine learning model labels were derived from these clinician-confirmed definitions to eliminate misclassification bias.

Ethical approval for the study was obtained from the Izmir Katip Celebi University Non-Interventional Clinical Research Ethics Committee (Decision No: 0497) and the Ege University Internal Medicine Department Academic Committee (Number: 54148036).

### Data collection

Data from Izmir Katip Celebi University Ataturk Training and Research Hospital were obtained via the hospital information system. Data from Ege University Hospital were collected in a prepared format by the physicians of the Department of Internal Medicine. The dataset comprised 45 variables collected from electronic health records. Demographic variables included individual patient identifier (ID), institution, age, and gender. Laboratory parameters consisted of neutrophil count (NEU), lymphocyte count (LYM), platelet count (PLT), mean platelet volume (MPV), albumin (ALB), C-reactive protein (CRP), blood urea nitrogen (BUN), creatinine (CRE), aspartate aminotransferase (AST), sodium (NA), partial thromboplastin time (PTT), international normalized ratio (INR), activated partial thromboplastin time (APTT), ferritin, D-dimer, fibrinogen, lactate dehydrogenase (LDH), procalcitonin, total bilirubin, and lactate. Clinical severity scores included the Acute Physiology and Chronic Health Evaluation II (APACHE-II) score, Sequential Organ Failure Assessment (SOFA) score, and Glasgow Coma Scale (GCS) score. Comorbidities recorded were chronic kidney disease, malignancy, diabetes mellitus, and hypertension. Clinical characteristics encompassed sepsis focus (categorized as thorax, urinary, blood-catheter, or other), heart rate, alteration in mental status, hypotension, hypoxemia, duration of intensive care unit stay, and hospital mortality. Additionally, binary variables were created to indicate plasma CRP exceeding 2 standard deviations from the normal value, plasma procalcitonin exceeding 2 standard deviations from the normal value, elevated creatinine, thrombocytopenia, hyperbilirubinemia, and elevated lactate levels.

### Data preprocessing

All variables were preprocessed prior to model development. Categorical features were transformed using one hot encoding to avoid imposing an artificial ordinal structure. Dummy variables were created for each categorical attribute. The encoding procedure was applied separately to the training and test sets, after which the test set was aligned with the training set to ensure identical feature structures. Missing categorical and continuous values were imputed using the HyperImpute framework, as detailed in the process for completing missing data. For continuous variables, z-score standardization was performed.

### Process for completing missing data

The dataset, comprising 1736 patients from two centers, contained an overall missing value rate of 19%. A variable-level summary of missingness is presented in Supplementary Tables 1, 2, which show that the extent of missing data varied across demographic, clinical, and laboratory variables. Examination of the missingness mechanism indicated that the data were consistent with a missing at random (MAR) pattern, as the likelihood of missingness was associated with observable patient characteristics. Missing values in both categorical and continuous variables were addressed using the HyperImpute imputation framework, which is widely utilized in healthcare settings for its ability to model complex nonlinear relationships among variables. HyperImpute operates by iteratively estimating each variable conditional on all others through flexible machine learning models, enabling coherent multivariate imputation. All imputation models were fitted on the training data and then were applied to the corresponding test data. All imputation procedures were implemented in the Google Colaboratory environment (Google Colaboratory environment; Google Compute Engine) (11).

### Feature selection procedure

In the feature selection process, the multicollinearity among the independent variables was assessed using the Variance Inflation Factor (VIF). No variable exhibited a VIF value exceeding the threshold of 10. However, the VIF values for the APACHE-II (5.74) and GCS (8.22) scores were notably higher than those of the other variables. Spearman correlation analysis was performed to identify highly correlated variable pairs. Variables exhibiting correlation coefficients exceeding 0.70 included: platelet count (PLT) and thrombocytopenia (ρ = −0.86), lactate and lactate elevation (ρ = 0.83), creatinine (CRE) and creatinine elevation (ρ = 0.82), APACHE-II score and GCS score (ρ = −0.79), GCS score and mental status alteration (ρ = −0.78), APACHE-II score and Sequential Organ Failure Assessment (SOFA) score (ρ = 0.78), and hyperbilirubinemia and total bilirubin (ρ = 0.78). To address multicollinearity while preserving clinically relevant variables, one variable from each highly correlated pair was retained based on clinical significance and study objectives. Specifically, PLT was retained over the binary thrombocytopenia indicator, lactate over lactate elevation, CRE over creatinine elevation, and total bilirubin over hyperbilirubinemia. For the scoring systems, APACHE-II and SOFA scores were retained due to their established clinical importance in sepsis assessment, while GCS was excluded because of its high correlation with both APACHE-II (ρ = −0.79) and mental status alteration (ρ = −0.78). Additionally, INR was excluded due to multicollinearity considerations. Variables not relevant to the study objectives, including individual patient identifier, institution, and hospital mortality, were also excluded from the feature set. Following this selection process, 35 variables were retained for machine learning model development.

### Statistical analysis

The demographic and clinical characteristics of the patients were examined using descriptive statistics via IBM SPSS Statistics 26.0 software. Descriptive statistics included sample size (*n*), percentage (*%*), mean ± standard deviation, median, interquartile range (*IQR*), and *p*-values. The data were analyzed using the Kolmogorov–Smirnov normality test. The Mann Whitney *U* test was employed to compare continuous variables that did not satisfy parametric test requirements. The dependency between categorical variables was assessed using the Continuity Correction and Pearson Chi-Square test. A *p*-value of less than 0.05 was considered statistically significant.

### Development and assessment of machine learning models

Machine learning analyses were conducted in the Google Colaboratory environment (Python 3 supported by Google Compute Engine) using 15 GB of RAM and a CPU processor. The dataset was split into training and testing sets at an 80:20 ratio. Model development was performed using the RandomForestClassifier, TensorFlow (Sequential, Dense, Dropout, Adam), XGBoost, and GaussianNB libraries. All models were trained with a fixed random seed (42). To evaluate model stability and generalizability, 10-fold cross-validation was applied within the training set. Feature importance was assessed for each model using classifier-derived rankings and SHapley Additive exPlanations (SHAP) values. SHAP analyses were performed using the shap and ShapModelInterpreter packages, and the top ten significant variables were reported.

The hyperparameters of each machine learning model were defined through manual search and are fully described to ensure reproducibility. For the Artificial Neural Network (ANN), the architecture consisted of three hidden layers with 64, 32, and 16 neurons, respectively, all using ReLU activation. Dropout regularization followed each hidden layer with dropout rates of 0.5, 0.4, and 0.3. The output layer contained a single sigmoid-activated neuron. The model was trained using the Adam optimizer (learning rate = 0.05), a batch size of 16, and a maximum of 50 epochs. Binary cross-entropy was selected as the loss function, and early stopping with a patience of 10 epochs was applied. For the Random Forest classifier, the model comprised 15 decision trees with a maximum depth of 2. A minimum of 2 samples was required to split an internal node, and at least 5 samples were required per leaf. The number of features available at each split was set to the square root of the total classifiers. The XGBoost model was trained with 22 boosting rounds, a maximum depth of 2, a learning rate of 0.05, and a minimum child weight of 10. Subsampling involved 10% of the training samples and 15% of features per tree. The gamma parameter was set to zero, while L1 and L2 regularization terms (alpha and lambda) were kept at their default values. The objective function was binary logistic regression, with log-loss used for evaluation. For the Naive Bayes classifier, default settings were retained, with no pre-defined class priors (priors = None), allowing class probabilities to be learned directly from the data. The var_smoothing parameter was maintained at its default value (1×10^−9^) to ensure numerical stability. The assessment of model performances provided sensitivity, specificity, false positive rate, false negative rate, positive predictive value, negative predictive value, accuracy, precision, F1 score, AUROC, and confusion matrix for each classifier.

**Figure 1:**
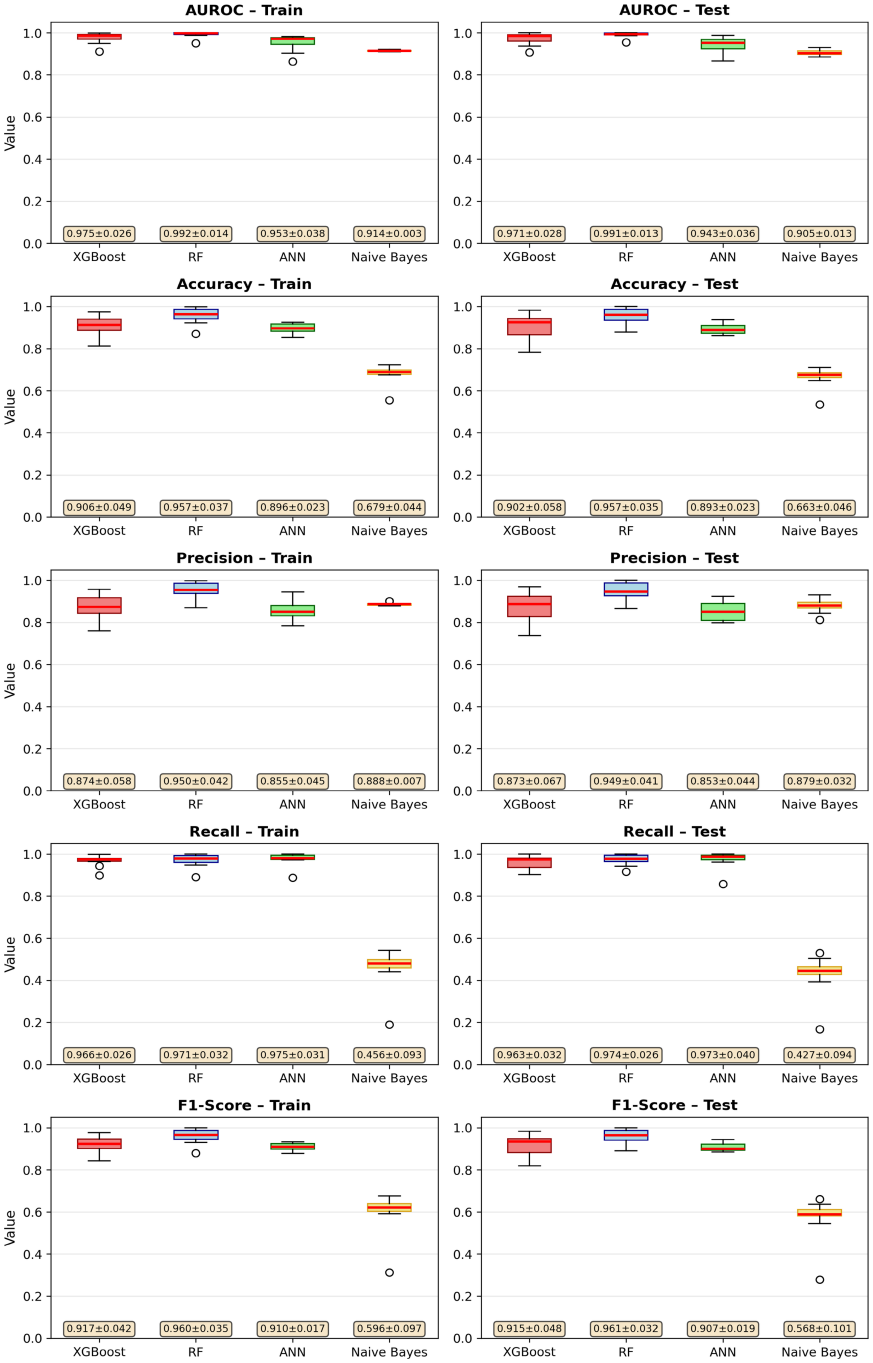
Training and test set performance comparison of all models using 10 random seeds.

To evaluate model generalizability, both training and test performance were systematically examined across accuracy, precision, recall, F1-score, and AUROC. Table 1 presents the training versus test metrics for all four classifiers using the primary train-test split (random seed = 42). The comparable values across datasets suggest that none of the models exhibit substantial overfitting or underfitting. In particular, XGBoost and Random Forest demonstrated nearly parallel performance patterns in both training and testing sets. To assess robustness beyond a single split, each model was additionally trained using 10 different random seeds (42, 123, 456, 789, 1,011, 1,314, 1,617, 1920, 2,223, and 2,526). The boxplots summarizing these repeated evaluations illustrate narrow performance distributions for XGBoost and Random Forest, indicating model stability and resistance to randomness in data partitioning while ANN and Naive Bayes exhibited wider yet clinically acceptable variability (Figure 1).

**Table 1:** Performance metrics of machine learning models.

Evaluation set	Models	Recall	Specificity	FPR	FNR	PPV	NPV	Accuracy	Precision	F1 Score	AUROC
Training set	RF	0.8752	0.8289	0.1711	0.1248	0.8574	0.8497	0.8539	0.8574	0.8662	0.9448
	ANN	0.9408	0.7838	0.2162	0.0592	0.8364	0.9185	0.8686	0.8364	0.8855	0.9445
	XGB	0.9616	0.7989	0.2011	0.0384	0.8489	0.9465	0.8868	0.8489	0.9017	0.9746
	NB	0.4864	0.9286	0.0714	0.5136	0.8889	0.6061	0.6897	0.8889	0.6287	0.9099
Test set	RF	0.9116	0.7762	0.2238	0.0884	0.8072	0.8952	0.8448	0.8072	0.8562	0.9343
	ANN	0.9116	0.7552	0.2448	0.0884	0.7929	0.8926	0.8345	0.7929	0.8481	0.9262
	XGB	0.9796	0.7762	0.2238	0.0204	0.8182	0.9737	0.8793	0.8182	0.8916	0.9750
	NB	0.4694	0.9091	0.0909	0.5306	0.8415	0.6250	0.6862	0.8415	0.6026	0.9034
Cross Validation	RF	0.8735	0.7951	0.2049	0.1265	0.8359	0.8441	0.8375	0.8359	0.8532	0.9358
	ANN	0.9705	0.8288	0.1712	0.0295	0.8695	0.9622	0.9032	0.8695	0.9151	0.9680
	XGB	0.9856	0.8232	0.1768	0.0144	0.8683	0.9797	0.9110	0.8683	0.9231	0.9860
	NB	0.4737	0.9154	0.0846	0.5263	0.8700	0.5985	0.6767	0.8700	0.6097	0.9000

RF, Random Forest; ANN, Artificial Neural Networks; XGB, eXtreme Gradient Boosting; NB, Naive Bayes; FPR, false positive rate; FNR, false negative rate; PPV, positive predictive value; NPV, negative predictive value.

Ten-fold cross-validation was applied to estimate the generalizability of each model on the training set. Cross-validation was not used for hyperparameter tuning. All hyperparameters were predetermined via manual search. The purpose of cross-validation in this study was to quantify variability across resampled subsets of the training data rather than to conduct model selection. Nested cross-validation was not implemented. Although this approach has been suggested as an alternative for small datasets, further work is required to explore its potential in future studies. The test set was held out prior to any training or validation procedures and remained completely independent from cross-validation. All reported test metrics were obtained by evaluating the final models on this unseen dataset.

## Results

### Comparison of demographic and clinical data

Table 2 presents demographic and clinical data on continuous quantitative characteristics comparing groups with and without thrombocytopenia in sepsis patients. Patients with thrombocytopenia were significantly younger than those without, with a notable age difference (*p* < 0.001). AST, APACHE II and SOFA scores, ferritin, LDH, procalcitonin, total bilirubin, and lactate levels were significantly higher in individuals with thrombocytopenia, with statistical significance (*p* < 0.001). Values of NEU, LYM, PLT, and fibrinogen were lower in patients with thrombocytopenia, with statistical significance (*p* < 0.001).

**Table 2:** Demographic and clinical characteristics of sepsis patients (continuous variables).

Variables	Total (*n* = 1,447)	Thrombocytopenia No (*n* = 675)	Thrombocytopenia Yes (*n* = 772)	*p* value
	Mean ± SD Median (IQR)	Mean ± SD Median (IQR)	Mean ± SD Median (IQR)	
Age^+^	63.85 ± 16.78 66.00 (22.00)	66.21 ± 15.87 68.00 (20.00)	61.79 ± 17.29 64.00 (24.00)	<0.001
NEU^+^	11253.94 ± 9496.97 9180.00 (9910.00)	13094.85 ± 9793.06 10710.00 (10230.00)	9644.33 ± 8930.58 7965.00 (10070.00)	<0.001
LYM^+^	1482.10 ± 3098.07 940.00 (970.00)	1479.45 ± 3103.28 1150.00 (1010.00)	1176.68 ± 3843.22 735.00 (907.50)	<0.001
PLT^+^	183160.33 ± 146109.77 141000.00(183000.00)	302961.48 ± 129224.06 270000.00(156000.00)	78411.92 ± 43454.83 78500.00 (80000.00)	<0.001
MPV^+^	10.95 ± 1.30 10.90 (1.70)	10.56 ± 1.23 10.50 (1.50)	11.28 ± 1.27 11.30 (1.50)	<0.001
ALB^+^	3.08 ± 3.00 2.79 (0.37)	3.03 ± 2.50 2.81 (0.23)	3.13 ± 3.38 2.76 (0.52)	<0.001
CRP^+^	115.94 ± 111.36 83.80 (165.73)	109.58 ± 107.30 78.39 (160.25)	121.51 ± 114.56 89.64 (172.54)	0.034
BUN^+^	54.82 ± 45.03 42.00 (51.00)	51.05 ± 46.94 38.00 (49.00)	58.10 ± 43.06 47.00 (52.00)	<0.001
CRE^+^	2.10 ± 1.80 1.43 (2.12)	2.11 ± 1.80 1.42 (2.20)	2.09 ± 1.81 1.43 (2.03)	0.868
AST^+^	154.09 ± 544.59 38.00 (58.00)	115.74 ± 425.75 35.00 (41.00)	187.62 ± 628.77 41.00 (78.00)	<0.001
NA^+^	138.31 ± 12.84 137.00 (9.00)	138.61 ± 16.71 137.00 (7.00)	138.04 ± 8.06 137.00 (9.00)	0.336
PTT^+^	16.82 ± 8.22 14.60 (4.20)	16.07 ± 8.54 14.20 (3.40)	17.47 ± 7.87 15.30 (5.05)	<0.001
APTT^+^	35.51 ± 17.53 31.90 (9.90)	32.93 ± 12.06 30.80 (8.10)	37.76 ± 20.94 33.05 (12.00)	<0.001
APACHE-II^+^	19.60 ± 4.74 19.00 (4.00)	18.29 ± 4.55 18.00 (4.00)	20.75 ± 4.60 20.00 (4.00)	<0.001
SOFA^+^	7.43 ± 2.07 7.00 (3.00)	6.54 ± 1.57 6.00 (1.00)	8.21 ± 2.15 8.00 (2.00)	<0.001
Ferritin^+^	3823.20 ± 4403.05 2481.00 (2921.00)	2684.91 ± 2991.01 1881.00 (1337.00)	4818.47 ± 5139.35 3288.50 (3842.30)	<0.001
D-Dimer^+^	2199.40 ± 3346.99 1387.00 (1211.00)	1965.95 ± 2272.55 1358.00 (828.00)	2403.53 ± 4050.38 1473.00 (2122.00)	0.611
Fibrinogen^+^	421.07 ± 97.53 419.00 (61.00)	436.75 ± 74.12 429.00 (38.00)	407.36 ± 112.39 405.00 (69.00)	<0.001
LDH^+^	489.53 ± 382.76 363.00 (106.00)	440.05 ± 307.07 350.00 (62.00)	532.79 ± 433.96 379.00 (140.00)	<0.001
ICU Duration^+^	15.90 ± 44.01 3.00 (14.00)	16.96 ± 51.35 3.00 (14.00)	14.98 ± 36.40 4.00 (15.00)	0.064
Procalcitonin^+^	21.02 ± 49.86 10.50 (21.25)	14.74 ± 29.61 8.59 (16.64)	26.51 ± 61.90 14.03 (25.07)	<0.001
T. Bilirubin^+^	2.35 ± 3.79 1.16 (1.60)	1.75 ± 2.95 0.96 (1.01)	2.87 ± 4.33 1.44 (2.53)	<0.001
Lactate^+^	3.89 ± 3.89 2.50 (2.80)	3.13 ± 3.16 2.10 (1.80)	4.56 ± 4.33 3.00 (3.50)	<0.001

NEU, neutrophil; LYM, lymphocyte; PLT, platelet; MPV, mean platelet volume; ALB, albumin; CRP, C-reactive protein; BUN, blood urea nitrogen; CRE, creatinine; AST, aspartate aminotransferase; NA, sodium; PTT, partial thromboplastin time; APTT, activated partial thromboplastin time; APACHE-II, acute physiological and chronic health assessment; SOFA, sequential organ failure assessment score; LDH, lactate dehydrogenase; ICU, intensive care unit; T, total; SD, standard deviation; Median (IQR, Interquartile deviation),^+^ Mann Whitney U test.

Table 3 presents demographic and clinical data on categorical characteristics comparing groups with and without thrombocytopenia in sepsis patients. No significant gender difference was seen between individuals with and without thrombocytopenia. The prevalence of thrombocytopenia was higher in men (about 54%). A statistically significant difference is found between patients with and without thrombocytopenia regarding malignancy, diabetes, hypertension, and hypotension results (*p* < 0.001). A statistically significant difference is found between patients with and without thrombocytopenia regarding sepsis focus, with thrombocytopenia occurring in 56.5% of patients with infections excluding thoracic, blood-catheter, and urinary tract infections (*p* = 0.003).

**Table 3:** Demographic and clinical characteristics of sepsis patients (categorical variables).

Variables	Total (*n* = 1,447)	Thrombocytopenia No (*n* = 675)	Thrombocytopenia Yes (*n* = 772)	*p* value
	*n* (%)	*n* (%)	*n* (%)	
Gender^**^				
Male	825 (57.0)	382 (46.3)	443 (53.7)	0.762
Female	622 (43.0)	293 (47.1)	329 (52.9)	
Chronic K. D. ^**^				
No	1,252 (86.5)	568 (45.4)	684 (54.6)	0.013
Yes	195 (13.5)	107 (54.9)	88 (45.1)	
Cancer^**^				
No	1,042 (72.0)	541 (51.9)	501 (48.1)	<0.001
Yes	405 (28.0)	134 (33.1)	271 (66.9)	
Diabetes^**^				
No	991 (68.5)	422 (42.6)	569 (57.4)	<0.001
Yes	456 (31.5)	253 (55.5)	203 (44.5)	
Hypertension^**^				
No	809 (55.9)	336 (41.5)	473 (58.5)	<0.001
Yes	638 (44.1)	339 (53.1)	299 (46.9)	
Sepsis Focus^**^				
Thorax	256 (17.7)	113 (44.1)	143 (55.9)	0.003
Urinary	172 (11.9)	75 (43.6)	97 (56.4)	
Blood-catheter	398 (27.5)	217 (54.5)	181 (45.5)	
Other	621 (42.9)	270 (43.5)	351 (56.5)	
Heart Rate^**^				
No	902 (62.3)	418 (46.3)	484 (53.7)	0.763
Yes	545 (37.7)	257 (47.2)	288 (52.8)	
Mental Status^**^				
No	1,051 (72.6)	506 (48.1)	545 (51.9)	0.063
Yes	396 (27.4)	169 (42.7)	227 (57.3)	
Plasma CRP > 2 SD from normal value^**^				
No	1,382 (95.5)	652 (47.2)	730 (52.8)	0.063
Yes	65 (4.5)	23 (35.4)	42 (64.6)	
Plasma procalcitonin >2 SD from normal value*				
No	1,426 (98.5)	673 (47.2)	753 (52.8)	0.001
Yes	21 (1.5)	2 (9.5)	19 (90.5)	
Hypotension^**^				
No	720 (49.8)	393 (54.6)	327 (45.4)	<0.001
Yes	727 (50.2)	282 (38.8)	445 (61.2)	
Hypoxemia^**^				
No	538 (37.2)	253 (47.0)	285 (53.0)	0.825
Yes	909 (62.8)	422 (46.4)	487 (53.6)	

K. D., kidney disease; CRP, C-reactive protein; SD, standard deviation; ^*^ continuity correction test; ^**^ Pearson Chi-Square test.

### Evaluation of machine learning methods

Table 1 displays the performance metrics of the machine learning models. The training set results indicate that XGBoost yielded the best overall training performance with the highest recall (96.16%), F1-score (90.17%), accuracy (88.68%), and AUROC (97.46%), together with high NPV (94.65%), indicating that it captured the majority of thrombocytopenia cases while maintaining good specificity (79.89%). Random Forest and ANN showed very similar and consistently strong results, with AUROC values of 94.48 and 94.45%, accuracies of 85.39 and 86.86%, and balanced precision-recall profiles (F1-scores 86.62 and 88.55%, respectively). Naive Bayes had the lowest recall (48.64%) and F1-score (62.87%), but the highest specificity (92.86%) and PPV (88.89%), correctly identifying non-thrombocytopenic patients at the expense of missing a substantial proportion of true positive cases. The test set results indicate that XGBoost achieved the highest performance, with an accuracy of 87.93% and an F1 score of 89.16%. In comparison, RF and ANN recorded accuracies of 84.48 and 83.45%, along with F1 scores of 85.62 and 84.81%, respectively. NB exhibited the poorest performance, achieving an accuracy of 68.62% and an F1 score of 60.26%. The cross-validation findings indicate that XGBoost achieved the highest performance, with an accuracy of 91.10% and an F1 score of 92.31%. In comparison, ANN and RF recorded accuracy of 90.32 and 83.75%, along with F1 scores of 91.51 and 85.32%, respectively. NB exhibited the poorest performance, achieving 67.67% accuracy and a 60.97% F1 score.

**Figure 2:**
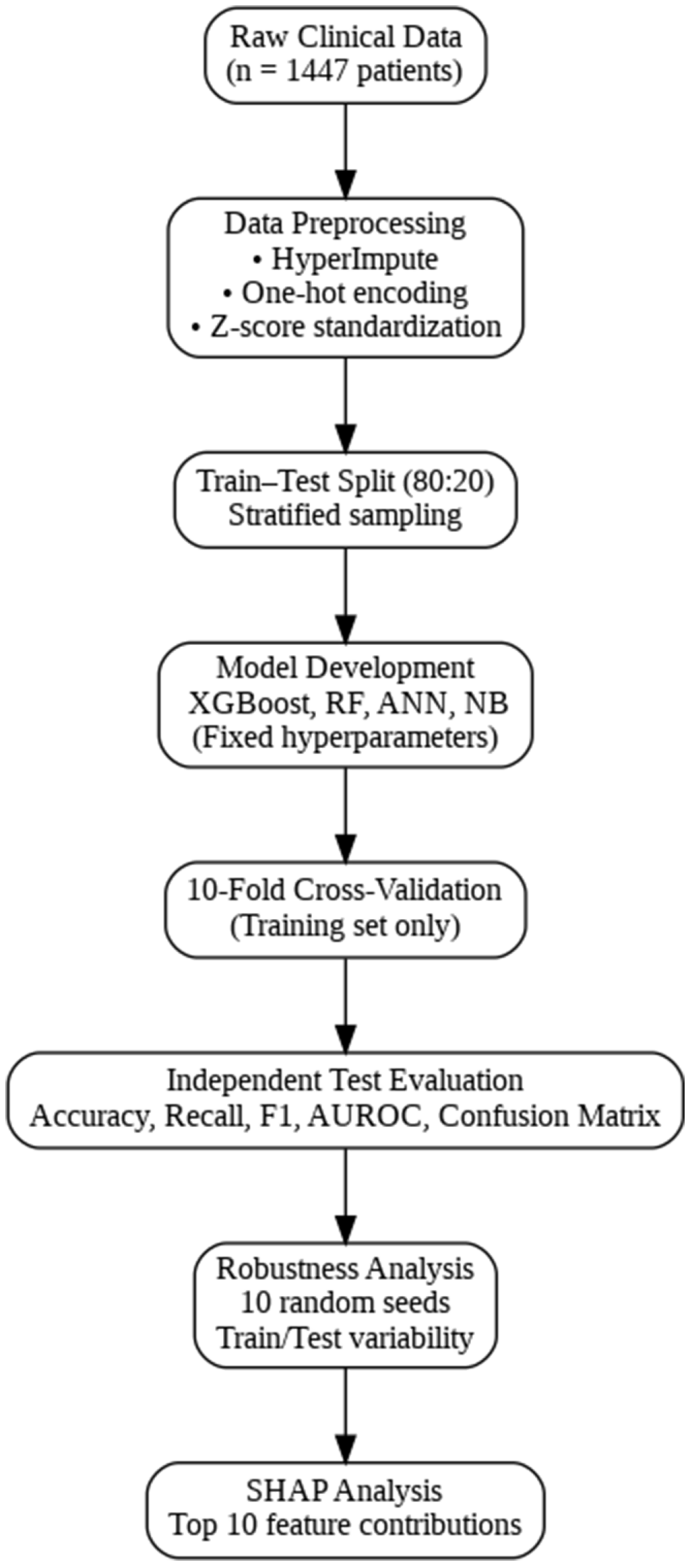
Workflow diagram.

Figure 2 displays the sequential stages employed to develop, validate, and interpret the machine learning models. Raw clinical data from 1,447 patients were initially preprocessed using HyperImpute for missing-data imputation, one-hot encoding for categorical variables, and z-score standardization for continuous variables. The dataset was then divided into training and test sets using an 80:20 stratified split. Four classifiers XGBoost, Random Forest, Artificial Neural Network, and Naive Bayes were trained using fixed hyperparameters. Model performance on the training data was assessed through 10-fold cross validation to evaluate internal generalizability. Independent evaluations on the unseen test set included accuracy, recall, precision, F1-score, AUROC, and confusion matrices. Robustness analyses were conducted by repeating the entire training–testing process using 10 different random seeds to assess performance variability across data partitions. Finally, SHAP-based explainability analyses were performed to determine the top contributing features and enhance model interpretability.

**Figure 3:**
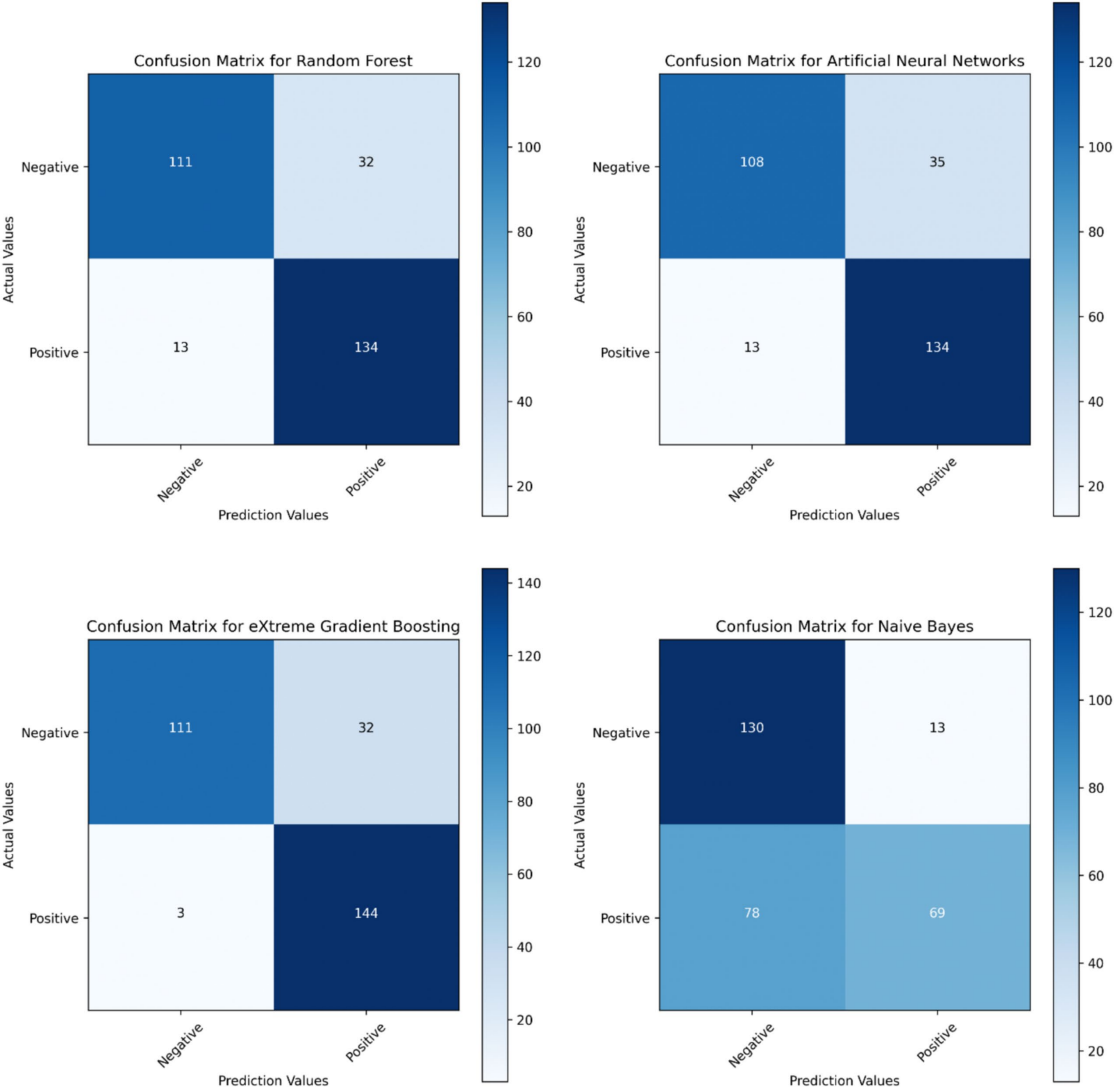
Confusion matrices of machine learning models.

Based on the confusion matrices of the machine learning models presented in Figure 3, the most effective model for identifying patients with thrombocytopenia as positive is XGBoost, utilizing 144 samples, whereas the most effective model for identifying patients without thrombocytopenia as negative is the NB model, employing 130 samples. RF identified 134 patients with thrombocytopenia as positive and 111 patients without thrombocytopenia as negative; ANN identified 134 patients with thrombocytopenia as positive and 108 patients without thrombocytopenia as negative, yielding comparable results.

Table 4 displays the top 10 significant features as determined by the classifiers of machine learning models. The significant variables shared among the four models were identified as platelet and lactate. RF, XGBoost, and NB analyses identified platelet and SOFA Score as the two primary variables.

**Table 4:** Top 10 important features in machine learning models.

Rank	RF Feature	%	ANN Feature	%	XGB Feature	%	NB Feature	%
1	SOFA Score	23.09	LYM	15.78	PLT	47.75	PLT	28.06
2	PLT	12.50	Gender	10.45	SOFA Score	16.46	SOFA Score	15.05
3	Ferritin	10.36	Age	10.42	Ferritin	7.30	MPV	10.46
4	Fibrinogen	10.35	NEU	9.65	Hypotension	5.35	APACHE-II	9.18
5	NEU	9.07	NA	9.60	Fibrinogen	4.71	Ferritin	8.89
6	LYM	8.84	PLT	8.89	APACHE-II	4.58	Lactate	6.64
7	Lactate	8.12	Lactate	8.87	Lactate	4.20	NEU	6.18
8	MPV	7.52	AST	8.83	T. Bilirubin	3.65	Fibrinogen	5.40
9	ALB	5.55	ICU Duration	8.77	Procalcitonin	3.37	APTT	5.15
10	D-Dimer	4.58	LDH	8.74	Age	2.62	T. Bilirubin	4.99

RF, Random Forest; ANN, Artificial Neural Networks; XGB, eXtreme Gradient Boosting; NB, Naive Bayes; NEU, neutrophil; LYM, lymphocyte; PLT, platelet; MPV, mean platelet volume; ALB, albumin; AST, aspartate aminotransferase; APTT, activated partial thromboplastin time; APACHE-II, acute physiological and chronic health assessment; SOFA, sequential organ failure assessment score; LDH, lactate dehydrogenase; ICU, intensive care unit; T, total.

The SHAP results of the machine learning models indicate that the top 10 significant features are presented in Table 5. The significant variables consistent throughout the SHAP results of the four models were identified as platelet count, SOFA score, fibrinogen, ferritin, and total bilirubin. The RF, ANN, and NB SHAP results identified platelet and SOFA score as two significant variables.

**Table 5:** Top 10 important features on SHAP results.

Rank	RF Feature	RF Feature (%)	ANN Feature	ANN Feature (%)	XGB Feature	XGB Feature (%)	NB Feature	NB Feature (%)
1	PLT	21.08	PLT	39.83	PLT	38.22	PLT	30.28
2	SOFA Score	16.61	MPV	13.18	Ferritin	9.77	SOFA Score	12.58
3	Fibrinogen	11.68	SOFA Score	10.31	Fibrinogen	9.65	Ferritin	9.90
4	Ferritin	9.20	NEU	9.17	Age	8.87	T. Bilirubin	9.39
5	NEU	8.80	T. Bilirubin	5.80	Lactate	7.47	Lactate	7.06
6	MPV	8.72	Ferritin	5.68	SOFA Score	6.97	ALB	6.89
7	Lactate	7.32	Hypotension	4.72	APACHE-II	6.94	APTT	6.51
8	LYM	6.39	Age	4.17	ICU Duration	4.63	Fibrinogen	6.50
9	ALB	5.48	Sepsis Focus (Blood-catheter)	4.02	Hypotension	4.20	Plasma CRP > 2 SD from normal value	5.45
10	T. Bilirubin	4.73	Fibrinogen	3.12	T. Bilirubin	3.30	Cancer	5.43

RF, Random Forest; ANN, Artificial Neural Networks; XGB, eXtreme Gradient Boosting; NB, Naive Bayes; NEU, neutrophil; LYM, lymphocyte; PLT, platelet; MPV, mean platelet volume; ALB, albumin; CRP, C-reactive protein; APTT, activated partial thromboplastin time; APACHE-II, acute physiological and chronic health assessment; SOFA, sequential organ failure assessment score; ICU, intensive care unit; T, total; SD, standard deviation.

**Figure 4:**
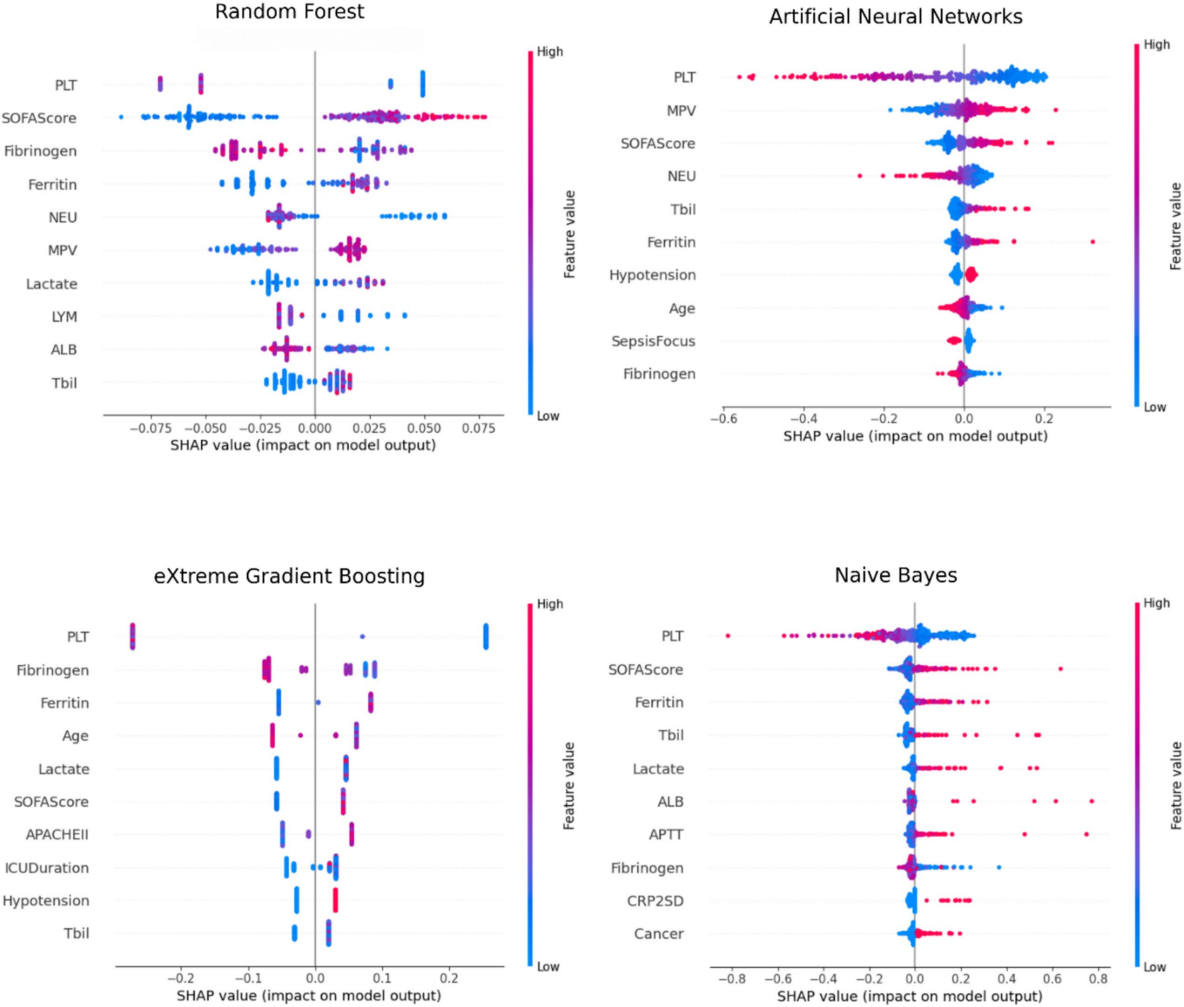
Summary plots of machine learning models based on SHAP results.

Figure 4 presents the SHAP summary plots of the machine learning models. The summary plot displays the rankings and effect distributions of characteristics significant in identifying thrombocytopenia in patients with sepsis. This means that, while the impact of thrombocytopenia is minimal in positive platelet numbers, the risk of thrombocytopenia escalates in negative values. Conversely, the effect of thrombocytopenia is pronounced in positive SOFA Score values, whereas the risk diminishes in negative values.

**Figure 5:**
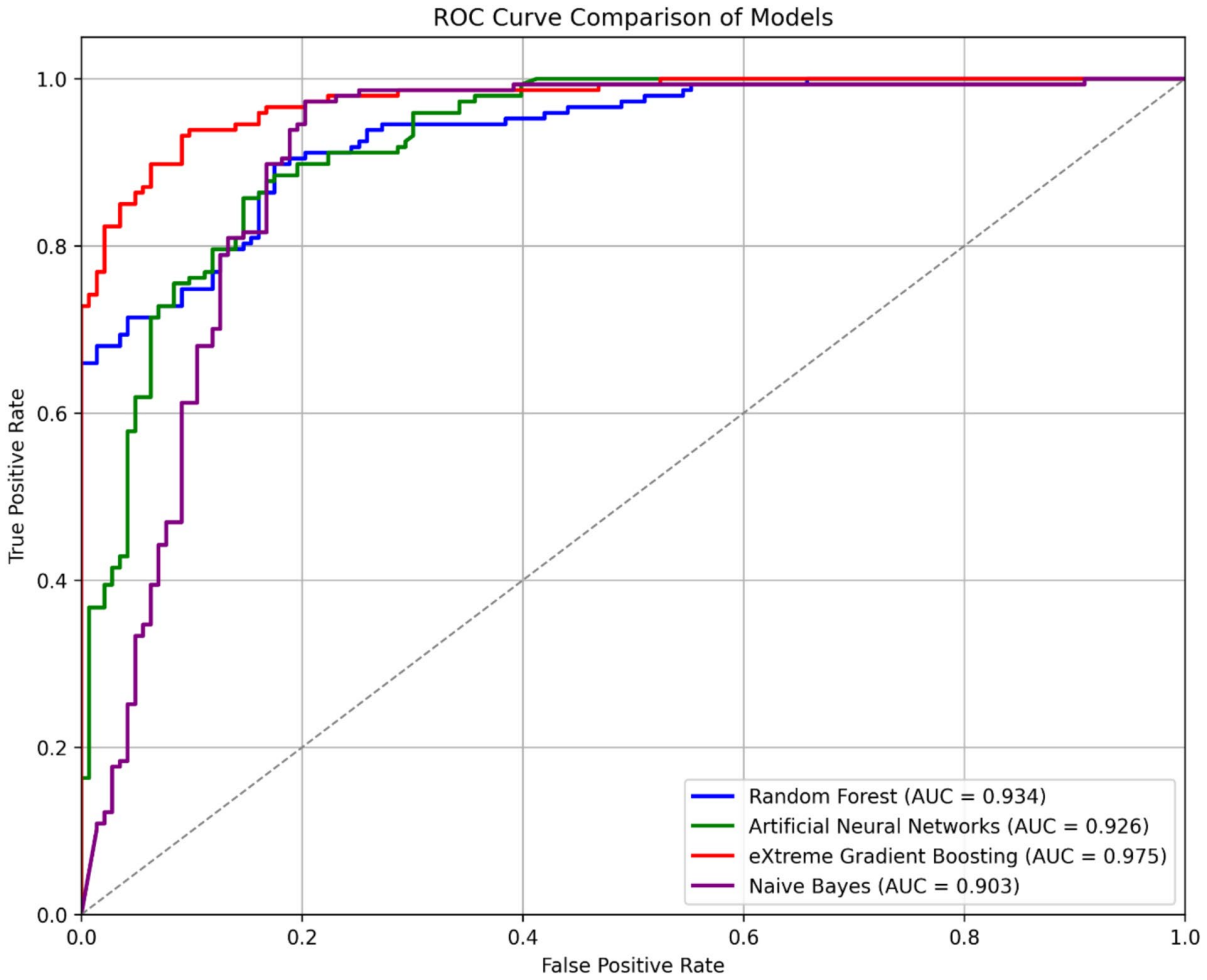
ROC curve comparison of machine learning models.

The XGBoost model, exhibiting an area under the ROC curve value of 97.5%, is identified as the most effective in distinguishing between positive and negative classes, as illustrated in Figure 5. The RF and ANN models achieve 93.4 and 92.6%, respectively, trailing the XGBoost model, while the NB model ranks lowest at 90.3%.

Figure 1 presents the robustness analysis performed across 10 independent train-test splits generated using distinct random seeds. For each split, all four classifiers were retrained and evaluated using identical preprocessing procedures and fixed hyperparameters. The figure depicts the distribution of AUROC, accuracy, precision, recall, and F1-score for both the training and test sets. Across these repeated evaluations, XGBoost and Random Forest demonstrated the highest degree of stability, showing narrow variability ranges and nearly overlapping performance distributions between training and test sets. ANN exhibited moderate variability but maintained consistent mean performance, whereas Naive Bayes showed greater fluctuation, particularly in recall, reflecting its sensitivity to class distribution across random splits. The robustness analysis confirms that the models, especially XGBoost and Random Forest generalize reliably under different data partitions, strengthening confidence in the validity and reproducibility of the results.

## Discussion

Platelets are crucial for hemostasis, and thrombocytopenia is associated with a poor prognosis in critical care unit patients (6, 12). Current research indicates that thrombocytopenia occurs in over 55% of sepsis patients, while our investigation found thrombocytopenia in 53.4% of sepsis patients (5).

This study assessed the performance of RF, ANN, XGBoost, and NB algorithms in SAT diagnosis. The comparison model performances indicated that the classification accuracy of the models exceeded 80% when assessed using the F1 criterion, while all models, except for NB, achieved 90% or higher when evaluated with the AUROC criterion.

XGBoost demonstrated superior performance in the cross-validation findings, achieving accuracy and AUROC scores of 91.10 and 98.60%, respectively. ANN (90.32%; 96.80%) and RF (83.75%; 93.58%) rank subsequent to the XGBoost model, respectively. NB ranked last, exhibiting the lowest accuracy and AUROC with 67.67 and 90.00%, respectively.

The test set findings indicate that XGBoost exhibited superior performance based on accuracy and AUROC metrics (87.93%; 97.50%), followed by RF (84.48%; 93.43%) and ANN (83.45%; 92.62%), respectively. NB ranked last, exhibiting the lowest accuracy and AUROC with 68.62 and 90.34%, respectively.

In diagnosing thrombocytopenia, the two most effective models based on test set accuracy were XGBoost (87.93%) and Random Forest (84.48%); however, cross-validation results indicated XGBoost (91.10%) and Artificial Neural Network (90.32%) as superior, while Naive Bayes exhibited the lowest accuracy (68.62, 67.67%). XGBoost excelled in the positive detection of thrombocytopenic individuals, whereas Naive Bayes was most effective in the negative detection of patients without thrombocytopenia.

The high recall performance of the XGBoost model warrants particular emphasis from a clinical perspective. In cross-validation, the model achieved a recall of 98.56, and 97.96% in the test set, indicating that it correctly classified nearly all true SAT-positive cases while minimizing false-negative results. This characteristic is critically important in sepsis management, where the consequences of a missed SAT diagnosis (false negative) are considerably more severe than those of a false-positive result. Undetected thrombocytopenia in septic patients can lead to delayed recognition of disease severity, inadequate monitoring of coagulation status, increased risk of hemorrhagic complications, and ultimately higher mortality rates. While false positive classification may lead to additional clinical investigation, it causes minimal harm and can encourage closer monitoring. Therefore, a model with high recall may facilitate the identification of a large proportion of at-risk patients, highlighting its potential relevance for future research in high-intensity clinical care settings where early recognition of SAT is clinically important.

Although this study evaluates machine learning models in cross-sectional diagnostic research, the results may offer insights relevant to future clinical research. In clinical practice, clinicians integrate laboratory measurements and physiological indicators obtained at admission or during the early stages of sepsis to assess disease severity and complication risk. Machine learning models such as XGBoost may provide a basis for developing decision support tools in the future. Any potential implementation within electronic health record systems would require external validation, standardized cohort definitions, careful variable harmonization, and recalibration across independent clinical settings. Following such validation, routinely collected laboratory parameters including platelet count, SOFA score, ferritin, fibrinogen, and related biomarkers could be explored as inputs for risk stratification models aimed at supporting, rather than replacing, clinical assessment. These approaches may be particularly relevant for research in advanced critical care environments, where early recognition of thrombocytopenia is clinically important. Any clinical application should be considered exploratory until prospectively validated across diverse populations.

Jiang et al. reported the highest performance for ANN with an AUROC of 79.0%, whereas in our research, XGBoost ranked first (97.5%), followed by RF and ANN (12). In the same study, RF exhibited the lowest performance at 74.0%, although in our study, NB demonstrated the lowest performance at 90.3% (12). The research by Setarehaseman et al. identifies biomarkers capable of classifying thrombocytopenia and severe thrombocytopenia in sepsis, including patient age, platelet count, MPV, CRP, procalcitonin, D-Dimer, fibrinogen, bilirubin, albumin, NEU, LYM, and the SOFA and APACHE-II scores. The variables were incorporated in the top 10 significant feature rankings of SHAP and model classifiers in our research (13).

Wang et al. and Setarehaseman et al. identified hematological and other malignant conditions as significant biomarkers among the risk factors classifying SAT. In our study, hypotension was incorporated into the XGBoost SHAP ranking alongside ANN and XGBoost classifiers, whereas cancer findings and CRP levels exceeding two standard deviations were included in the NB SHAP ranking (10, 13). The classifier of four models identifies platelet and lactate as significant variables. In the research by Jiang et al., the SOFA Score is a critical variable in four models, and in our study, it is incorporated based on the findings from RF, XGBoost, and NB (12). SHAP results indicate that the shared significant variables across four models were platelet count, SOFA Score, fibrinogen, ferritin, and total bilirubin. In contrast, RF, ANN, and NB SHAP results identified platelet count and SOFA Score as the primary variables. In the research by Ling et al., the shared variables between the significant XGBoost SHAP variables and our study were the SOFA Score, lactate levels, and age (14).

The APACHE-II and SOFA scores, identified as significant risk factors for thrombocytopenic patients in the study by Coskun et al. and for septic patients in the research by Luka et al., are also critical variables in our research (15, 16). Procalcitonin, which forecasted short-term death in sepsis patients in the research by Luka et al., was the sole meaningful feature in the XGBoost classifier in our study (15). Semeraro et al. identified fibrinolysis and D-Dimer as critical markers for predicting death in sepsis patients; in our study, these were the only important variables within the RF classifier (17).

The study shows that machine learning algorithms possess considerable potential for identifying sepsis associated thrombocytopenia and relevant biomarkers. This study has some limitations. Although a multicenter dataset and a robust internal validation strategy were employed, external validation was not performed, representing a fundamental limitation. Variations in case mix, laboratory measurement practices, variable availability, data collection timing, and diagnostic labeling across institutions and healthcare systems may substantially influence model performance in independent clinical settings. Despite the inclusion of two centers, the sample size remains modest for machine learning research, and missing data were present across multiple clinical and laboratory variables. Advanced multivariate imputation using HyperImpute was applied to minimize information loss. Imputation procedures may introduce uncertainty and potential bias. The broad inclusion criteria and limited exclusion criteria, selected to capture a heterogeneous real world sepsis population, may have increased patient variability and further constrained generalizability. External validation using independent critical care databases was not undertaken, due to heterogeneity in sepsis-associated thrombocytopenia definitions, inconsistencies in variable definitions and measurement timing, and limited availability of key predictors across publicly available datasets. External validation conducted without cohort harmonization and variable matching may yield misleading performance estimates instead of accurately reflecting model generalizability.

## Conclusion

RF, ANN, XGBoost, and NB models were evaluated for sepsis-associated thrombocytopenia classification, together with SHAP-based interpretability analyses. Overfitting was mitigated through the use of an independent test set, 10-fold cross-validation, and early stopping strategies. The results demonstrate that machine learning models can effectively capture diagnostic patterns associated with SAT and identify relevant biomarkers. Among the evaluated algorithms, XGBoost demonstrated the strongest performance metrics within internal validation. Feature importance analyses across model classifiers consistently highlight platelet count and lactate levels as key variables. SHAP analyses further identified platelet count, SOFA score, fibrinogen, ferritin, and total bilirubin as shared influential features across models, underscoring their potential relevance as SAT associated biomarkers. When comparing model-based rankings and SHAP results, SHAP provides a broader and more coherent representation of shared influential features, along with intuitive visualizations that facilitate interpretation of feature effects on risk estimation. Future research should focus on prospective study designs, larger multicenter datasets, and diverse patient populations, with particular emphasis on standardized external validation to evaluate model generalizability and clinical relevance prior to any consideration of clinical application.

## Data Availability

The datasets presented in this article are not readily available because access to the data is limited to authorized researchers as approved by the participating institutions and their ethics committees. Due to these privacy and confidentiality regulations, the dataset is not publicly available but can be obtained from the corresponding author upon reasonable request and with appropriate institutional approvals. Requests to access the datasets should be directed to busra.emir@ikcu.edu.tr.
