# Supplementary material for: XGBoost outperforms other machine learning models in diagnosing Sepsis-Associated Thrombocytopenia: a multicenter retrospective study
Source: Front Med (Lausanne). 2026 Feb 9;13:1715551. doi: 10.3389/fmed.2026.1715551 (PMC12926416; doi:10.3389/fmed.2026.1715551)
Supplement: Supplementary file 1 [file Table_1.docx]

**Supplementary Table 1.** Missingness percentage of continuous variables

| **Variables** | Missing %  Total | Missing %  No | Missing %  Yes |
| --- | --- | --- | --- |
| Age | 0.00 | 0.00 | 0.00 |
| NEU | 3.15 | 0.92 | 5.38 |
| LYM | 3.09 | 0.23 | 5.96 |
| PLT | 0.00 | 0.00 | 0.00 |
| MPV | 19.56 | 22.29 | 16.84 |
| ALB | 42.60 | 43.92 | 41.29 |
| CRP | 1.67 | 1.83 | 1.52 |
| BUN | 0.00 | 0.00 | 0.00 |
| CRE | 0.00 | 0.00 | 0.00 |
| AST | 4.38 | 5.73 | 3.04 |
| NA | 0.92 | 1.03 | 0.82 |
| PTT | 4.38 | 5.96 | 2.81 |
| APTT | 24.68 | 29.84 | 19.53 |
| APACHE-II | 42.5 | 43.96 | 41.04 |
| SOFA | 52.85 | 49.85 | 55.86 |
| Ferritin | 38.57 | 41.27 | 35.88 |
| D-Dimer | 67.90 | 73.17 | 62.63 |
| Fibrinogen | 52.96 | 54.04 | 51.89 |
| LDH | 75.44 | 74.75 | 76.14 |
| ICU Duration | 0.00 | 0.00 | 0.00 |
| Procalcitonin | 39.15 | 45.09 | 33.22 |
| T. Bilirubin | 13.58 | 19.32 | 7.84 |
| Lactate | 29.07 | 25.27 | 32.88 |

*NEU:* Neutrophil, *LYM:* Lymphocyte, *PLT:* Platelet, *MPV:* Mean platelet volume, *ALB:* Albumin, *CRP:*C-reactive protein, *BUN:* Blood urea nitrogen, *CRE:* Creatinine, *AST:* Aspartate Aminotransferase,, *NA: S*odium, *PTT*: Partial thromboplastin time, *APTT: A*ctivated partial thromboplastin time, *APACHE-II:* Acute Physiological and Chronic Health Assessment, *SOFA:* Sequential Organ Failure Assessment Score, *LDH:* Lactate Dehydrogenase, *ICU:* Intensive care unit, *T:* Total
