# Supplementary material for: XGBoost outperforms other machine learning models in diagnosing Sepsis-Associated Thrombocytopenia: a multicenter retrospective study
Source: Front Med (Lausanne). 2026 Feb 9;13:1715551. doi: 10.3389/fmed.2026.1715551 (PMC12926416; doi:10.3389/fmed.2026.1715551)
Supplement: Supplementary file 2 [file Table_2.docx]

**Supplementary Table 2.** Missingness percentage of categorical variables

| **Variables** | Missing %  Total | Missing %  No | Missing %  Yes |
| --- | --- | --- | --- |
| Gender | 0.00 | 0.00 | 0.00 |
| Chronic Kidney Disease | 0.00 | 0.00 | 0.00 |
| Cancer | 0.00 | 0.00 | 0.00 |
| Diabetes | 0.00 | 0.00 | 0.00 |
| Hypertension | 0.00 | 0.00 | 0.00 |
| Sepsis Focus | 0.00 | 0.00 | 0.00 |
| Heart Rate | 0.00 | 0.00 | 0.00 |
| Mental Status | 64.10 | 63.65 | 64.56 |
| Plasma CRP >2 SD from normal value | 1.675 | 1.83 | 1.52 |
| Plasma procalcitonin >2 SD from normal value | 70.15 | 65.0.009 | 75.22 |
| Hypotension | 0.00 | 0.00 | 0.00 |
| Hypoxemia | 0.00 | 0.00 | 0.00 |
| Overall Missingness % | 18.64 | 19.11 | 18.17 |
